# Prevalence and Factors Associated with Meconium-Stained Amniotic Fluid in a Tertiary Hospital, Northwest Ethiopia: A Cross-Sectional Study

**DOI:** 10.1155/2021/5520117

**Published:** 2021-05-26

**Authors:** Enyew Abate, Kassahun Alamirew, Eleni Admassu, Awoke Derbie

**Affiliations:** ^1^Department of Obstetrics and Gynecology, College of Medicine and Health Sciences, Bahir Dar University, Bahir Dar, Ethiopia; ^2^School of Public Health, College of Medicine and Health Sciences, Bahir Dar University, Bahir Dar, Ethiopia; ^3^Department of Medical Laboratory Sciences, College of Medicine and Health Sciences, Bahir Dar University, Bahir Dar, Ethiopia; ^4^Center for Innovative Drug Development and Therapeutics Trail for Africa (CDT-Africa), Addis Ababa University, Addis Ababa, Ethiopia; ^5^Department of Health Biotechnology, Biotechnology Research Institute, Bahir Dar University, Bahir Dar, Ethiopia

## Abstract

*Background*. Fetal bowel could pass meconium, a green viscous fluid, before or during labour and most intrauterine passage of meconium is associated with several fetomaternal factors that lead to increased risk of perinatal morbidity and mortality. Given that there is a paucity of data, this study was conducted to assess the proportion and associated factors of meconium-stained amniotic fluid (MSAF) in women who came for labour and delivery service in a tertiary hospital. Methods. A cross-sectional study was conducted from 1 June to 31 August 2018 among 606 labouring mothers at Felege Hiwot Referral Hospital, northwest Ethiopia. Study participants were selected using a systematic random sampling technique. Data were collected using an interviewer-administered pretested questionnaire and data checklist. Factors associated with MSAF were explored using multivariable logistic regression analysis. Results. MSAF occurred in 24.6% (149/606) of pregnancies. Nonreassuring fetal heart rate patterns (Adjusted Odds Ratio [AOR]: 21.9, 95% Confidence interval [95% CI]: 10.96–43.83), postterm pregnancy (AOR: 4.54, 95% CI: 2.24–9.20), duration of labour more than 15 hours (AOR: 2.83, 95% CI: 1.76–4.53), pregnancy-induced hypertension (AOR: 2.43, 95% CI: 1.45–4.05), oligohydramnios (AOR: 2.53, 95% CI: 1.25–5.12), interpregnancy interval less than 2 years (AOR: 2.24, 95% CI: 1.12–4.51), and monthly family income less than 5000 Ethiopian Birr (185 USD) (AOR: 2.03, 95% CI: 1.18–3.51) were significantly associated with MSAF. Conclusions. In this study, the proportion of MSAF was at 24.6% which was higher than a previous report in Ethiopia. Nonreassuring fetal heart rate pattern, postterm pregnancy, duration of labour more than 15 hours, pregnancy-induced hypertension, oligohydramnios, interpregnancy interval less than 2 years, and monthly family income less than 5000 Ethiopian Birr were factors associated with an increased risk for MSAF. Therefore, interventions aimed at detecting MSAF early should consider these factors.

## 1. Background

Fetal bowel could pass meconium, a green viscous fluid, before or during labor and most intrauterine passage of meconium is associated with several fetomaternal outcomes that may lead to increased risk of perinatal morbidity and mortality [1–6]. MSAF can be found in 10–20% of laboring mothers across the globe [7–10], and a hospital-based study conducted in Jimma, Ethiopia, showed that 15.4% of women had MSAF [4].

A population-based cohort study conducted among women who gave birth to small for gestational age (SGA) neonates in Israel showed that gestational age at delivery, maternal age, and nonprogressive first stage of labor were all found to be independently associated with MSAF. SGA neonate with MSAF was independently associated with fetal distress [11]. A study in Carolina, USA, also showed an increased risk of MSAF for advancing gestational age, fetal stress, fewer than five prenatal care visits, and >15 hours of labor [12].

However, a prospective case-control study conducted in a tertiary-care hospital in India demonstrated that gestational age and parity showed no difference between the study and control groups. However, preeclampsia (32% vs. 6%), fetal growth restriction (12% vs. 2%), fetal distress (36% vs. 6%), and labor dystocia (20% vs. none) were found to be statistically different [13]. Maternal age and parity did not influence MSAF but MSAF was more common in pregnancies with pregnancy-induced hypertension, hepatitis, and severe anemia [14]. Maternal age >30 years, postdate pregnancy, cord problems, fetal distress, primgravidity, fetal growth restriction, gestational age ≥41 weeks, and women on oxytocin during labor were found associated with MSAF in other studies [15, 16].

Studies conducted arround the world showed difference in the proportion of MSAF with different determinant factors [16]. A majority of the studies conducted were in developed countries and middle-income countries [11, 12, 15, 16], and very little is known about the situation in, Ethiopia. Therefore, this study was conducted to assess the proportion and associated factors of MSAF at a tertiary hospital setup in Ethiopia.

## 2. Methods and Materials

### 2.1. Study Setting and Design

This was an institutional hospital-based cross-sectional study conducted in the period of 1 June to 31 August 2018 at Felege Hiwot Referral Hospital (FHRH), Bahir Dar, northwest Ethiopia. According to the Amhara Regional Health Bureau report, FHRH serves as a referral hospital for South Gondar, East and West Gojjam, Awi Zone, and Benshangul Gumuz Region. The hospital had 400 beds and 9 operating tables serving for more than 7 million populations in its catchment area.

The Department of Obstetrics and Gynecology had one big maternity ward with 74 beds for high-risk mothers, two operating tables, nine beds in the labor ward (seven beds in the first-stage room and two second-stage coaches), and seventeen postnatal and recovery beds. There were about 6000 deliveries per year and around 500 deliveries each month. The ward was organized with obstetricians and gynecologists, postgraduate residents, interns, clinical nurses, and midwives working as a care giver of the obstetrics service. The study area was selected because FHRH was the regional referral hospital and can be real representative for the population.

### 2.2. Study Population and Sample Size

All women who came for labor and delivery service to FHRH were our source population, and all mothers who gave birth at FHRH during the study period were considered the study population. The sample size was calculated with the single population proportion formula, at 95% CI, 3% marginal error [17], and using the proportion of MSAF conducted in Jimma, Southwest Ethiopia, at 15.4% [4, 18]. Adding 10% nonresponse rate, the final sample size was at 612.

### 2.3. Inclusion and Exclusion Criteria

All women with singleton pregnancy, gestational age 28 weeks and above, and who had delivered at FHRH were included in the study. Women who had breech presentation, pregnancy with unknown gestational age, fetal death upon admission, and fetal congenital malformations were excluded from the study.

### 2.4. Sampling and Sampling Procedure

A systematic random sampling technique was used to select the required number of participants. To sample our study participants, first, the average numbers of women who gave birth during the study period were estimated and obtained based on the number of deliveries over three months prior to data collection by referring to the delivery registration book/record. Totally, 1674 women delivered in three months with 558 deliveries per month. Secondly, so as to find the sampling fraction, the total number of women who delivered in three months (1674) was divided by the total number of our sample size (612) which was approximately 3. Thirdly, mothers who were admitted in the labor ward for labor and delivery are registered on the delivery registration log book and every third woman was included in the study and the first woman was selected using lottery method. During the sampling procedure, if the selected woman did not fulfill the inclusion criteria, the next woman was taken. 

### 2.5. Data Collection Tools and Analysis

Data were collected using the data checklist from maternal medical charts and interviewer-administered questionnaire by trained five BSc midwives supervised by two final-year residents. The interviewer-administered questionnaire and the data check list were prepared from different kinds of related literature which was structured into two groups (sociodemographic characteristics and previous and current obstetrics and medical history). Data on the sociodemographic characteristics and some of obstetrics-performance-related variables (gravidity, parity, gestational age, interpregnancy interval, and previous cesarean scar) and past medical history such as chronic hypertension and type 1 or type 2 diabetes mellitus were collected using the interviewer-administered questionnaire upon maternal admission to the labor ward, and data on some of obstetrics-related variables (status of the amniotic fluid, antepartum hemorrhage, premature rupture of membranes, pregnancy-induced hypertension, gestational diabetes mellitus, Fetal Growth Restriction (FGR), oligohydramnios, polyhydramnios, nonreassuring fetal heart rate pattern (NRFHRP), labor duration >15 hrs, labor induction, or augmentation), the presence or absence of maternal anemia, and newborn status were obtained after the woman gave birth using the data checklist from maternal follow-up charts, medical record charts, laboratory investigations, and delivery registration logbook throughout the course of labor until delivery and from neonatal intensive-care registration logbook and neonatal chart following neonates referred to the neonatal intensive-care unit.

### 2.6. Operational Definitions

Meconium-stained amniotic fluid (MSAF) is the presence of yellow, brownish, or green particulate matter in the amniotic fluid of a laboring mother until delivery of the baby. The final-year resident assigned to the labor ward had determined the presence or absence of MSAF throughout the course of labor.

Duration of labor: it was calculated by adding the number of hours the woman was laboring before admission that was taken from her history and the time she stayed in the hospital until delivery. Labor duration more than 15 hours was adopted from other literature as prolonged labor.

Nonreassuring fetal heart rate pattern (NRFHRP) was defined as fetal heart rate of <120 or >160 beats per minute that stays for more than 15 minutes using intermittent auscultations.

Maternal anemia is defined if the maternal hemoglobin level is less than 11 gram/deciliter.

Gestational age was calculated using the last menstrual period and or fetal biometric measurements performed by ultrasound before 22 weeks of gestational age.

Fetal growth restriction was diagnosed if symphysis fundal height was four weeks less than the gestational age and/or ultrasound diagnosis and/or postdelivery diagnosis of small for gestational age.

### 2.7. Data Management and Analysis

Data were entered and cleaned using Epi-data version 3.1 and transported to SPSS version 24 where analysis was conducted. Descriptive analyses such as frequencies and cross tabulations were performed. Multiple logistic regressions were fitted for MSAF, and odds ratio (OR) with their 95% confidence interval (95% CI) was calculated to identify the associated factors of meconium-stained amniotic fluid.

Variables with *p* values ≤0.2 remained in the model as potential confounders for multivariable analysis. Multivariable analysis was performed with the Hosmer–Lemeshow goodness-of-fit test with 95% confidence interval. Upon analysis, a backward conditional selection method was carried out until all the remaining variables were found to be significant with a *p* value of <0.05.

## 3. Results

A total of 1836 women gave birth during the study period at Felege Hiwot Referral Hospital, 606 women participated in the study, and 6 women were not interested with the study making the total response rate 99.1%.

### 3.1. Sociodemographic Characteristics

Among the study participants, the majority of women were in their 20s. Most resided in urban areas, married, and Orthodox Christian (Table 1).

### 3.2. Previous and Current Obstetric and Medical History

Among laboring women participated in the study, a majority of the pregnancies were planned, wanted, and supported. Most women were multiparous and had antenatal-care follow-up. Nearly half of study participants had antepartum obstetrics complications, and pregnancy-induced hypertension was the leading one (Table 2).

### 3.3. Proportion of Meconium-Stained Amniotic Fluid

The proportion of MSAF was at 24.6% [95% CI: 21.3–28.2%]. Early-Onset Neonatal Death (ENND), first- and fifth-minute Apgar score <7, neonatal resuscitation and intubation, and admissions to the Neonatal Intensive-Care Unit (NICU) were higher in newborns who were delivered with MSAF than their countertypes. NICU admissions for newborns with meconium-stained amniotic fluid were most often found to have meconium aspiration syndrome (55.6%), early-onset sepsis (14.8%), and perinatal asphyxia (8.6%) (Table 3).

### 3.4. Factors Associated with Meconium-Stained Amniotic Fluid

In this study, the association between demographic, obstetrical, medical history, and MSAF was assessed. The variables which showed an association at the bivariable analysis were client educational status, monthly income, parity, gestational age, antenatal-care status, premature rupture of the membranes, oligohydramnios, postterm pregnancy, fetal growth restriction, pregnancy-induced hypertension, previous CS scar, previous history of perinatal death, interpregnancy interval less than 2 years, maternal anemia, induction and augmentation of labor, NRFHRP (nonreassuring fetal heart rate pattern), and duration of labor >15 hours. These variables were taken for multivariable analysis to adjust for confounding factors. The Adjusted Odds Ratio (AOR) revealed that the presence of nonreassuring fetal heart pattern, postterm pregnancy, duration of labor more than 15 hours, pregnancy-induced hypertension, oligohydramnios, monthly income of the family, and interpregnancy interval less than 2 years had a significant association with MSAF.

The odds of having MSAF among women whose monthly family income was <5000 Ethiopian Birr (ETB) were 2.03 (95% CI: 1.18–3.51) times more likely than among women whose monthly income was 5000 ETB or more. Similarly, the occurrence of MSAF in women who had nonreassuring fetal heart rate pattern (NRFHRP) was 21.9 (95% CI: 10.96–43.83) times more likely than among women who did not have NRFHRP. Likewise, the odds of the presence of meconium-stained liquor in women with postterm pregnancy were 4.54 (95% CI: 2.24–9.20) times more likely than those who did not have postterm pregnancy, and women who labored for more than 15 hours were 2.83 (95% CI: 1.76–4.53) times more likely than those women whose labor had stayed less than 15 hours (Table 4).

## 4. Discussion

The proportion of MSAF in this study was at 24.6% [95% CI: 21.3–28.2%]. This study finding was higher than previous studies conducted in Israel (10.9%), India (8.3%), Nigeria (20.4%) and Jimma (15.4%), Bahir Dar, Ethiopia (17.8%) [4, 7–10, 19, 20]. This might be as a result of difference in study design, setup, and population. For instance, the study in Israel used a retrospective design among women with low-risk pregnancies, and the one in India also had different incidences within the same population. However, another study conducted in India, Kolkata, reported MSAF at 30.6% which was relatively higher than that in our study. The difference might be difference in the population; this study included only term pregnancies [21], but in our study, preterm women were also included.

With regard to factors associated with MSAF, monthly income of the family was one factor associated with the presence of MSAF in laboring mothers. Those women whose monthly income was less than 5000 ETB (185 USD) were 2 times more likely to develop MSAF than those whose income was 5000 ETB (185 USD) and above. This might be explained by fact that women who have low income tend to have delay in seeking medical care, tend to use public transport, or may came later when labor prolongs [22]. The presence of oligohydramnios was another factor associated with occurrence of MSAF. In this study, those women who had oligohydramnios were 2.5 times more likely to have meconium-stained liquor than those who did not have oligohydramnios. Similarly, studies conducted in India showed that oligohydramnios was significantly associated with MSAF [5, 20, 23]. Fetuses with oligohydramnios usually had low or inadequate energy reserve, so when women go into labor, there will be fetal intolerance to labor which will be manifested by passage of meconium as a result of hypoxia [24].

In our study, women with postterm pregnancy were 4.5 times more likely to have passage of meconium than women with term and preterm pregnancies. Studies conducted across the globe including USA, India, Brazil, Israel, Iran, China, and Australia showed similar findings (12, 15, 16, and 25–28). This might be explained by the maturation of the gastrointestinal tract and increased secretion of motilin by the fetus as gestational age advances which leads to increased fetal bowel peristalsis ending up in the passage of meconium [25, 26].

Pregnancy-induced hypertension was another factor associated with MSAF, and fetuses of women with this disorder were 2.4 times more likely to pass meconium than fetuses of those women who did not have this disorder. This result is consistent with other studies (5, 13). The association of pregnancy-induced hypertension with meconium passage is caused by uteroplacental insufficiency which causes fetal hypoxia leading to passage of meconium [27].

Women who labored for more than 15 hours were 2.8 times more likely to develop MSAF than those women who labored for less than 15 hours. This study was similar with other studies conducted in Lithuania, India, and South Korea [13, 28, 29]. The possible explanation for this might be an increased level of corticotrophin-releasing hormone resulting in increased cortisol during labor which is a known mediator of colonic motility resulting in meconium passage [30, 31]. Women with nonreassuring fetal heart rate pattern (NRFHRP) were nearly 22 times more likely to have meconium-stained liquor. Similarly, this finding was consistent with studies conducted in Nigeria, India (Kolkata), and the United States of America [10, 12, 15, 21, 32]. The explanation is oftentimes NRFHRP is a sign of hypoxia and hypoxia stimulates arginine vasopressin (AVP) release from the fetal pituitary gland and AVP stimulates colonic smooth muscle to contract, resulting in intra-amniotic defecation [24].

The last factor associated with development of MSAF was interpregnancy interval less than 2 years. Women whose interpregnancy intervals less than 2 years were 2.2 times more likely to develop meconium-stained liquor than others. As it is also stated in a previous similar study, this might be explained by the fact that women who had short interpregnancy interval might likely develop preeclampsia, increased risk fetal, and neonatal death [33].

This was a single site study conducted in a referral hospital only for three months because of time constraint, and generalization could be compromised because of seasonal variations and referral of patients with complications. No direct studies were found to compare the association of meconium-stained liquor with monthly income and interpregnancy interval less than 2 years. However, because of the availability of quite limited data in this area in Ethiopia, our study will be an important input for additional studies in the future.

## 5. Conclusions

The proportion of MSAF was at 24.6% which was higher than in a previous study conducted in Ethiopia. Nonreassuring fetal heart rate pattern, postterm pregnancy, duration of labor more than 15 hours, pregnancy-induced hypertension, oligohydramnios, interpregnancy interval less than 2 years, and monthly family income less than 5000 Ethiopian Birr were factors associated with an increased risk for MSAF. Health professionals should assess laboring women for the presence of these factors and should stay alert to detect MSAF early for better fetal and maternal heath outcomes.

## Figures and Tables

**Table 1:** Sociodemographic characteristics of study participants in Felege Hiwot Referral Hospital from June–August 2018 (*n* = 606).

Variables	Category	Number	Percent
Age in years	<19	28	4.6
	20–24	160	26.4
	25–29	229	37.8
	30–34	117	19.3
	>35	72	11.9

Place of residence	Urban	421	69.5
	Rural	185	30.5

Current marital status	Married	597	98.5
	Unmarried	9	1.5

Religion	Orthodox Christian	547	90.3
	Others	59	9.7

Client educational status	Cannot read and write	174	28.7
	Primary school	154	25.4
	Secondary school	123	20.3
	College or university	155	25.6

Client's occupation	Government employee	103	17
	Self-employed	35	5.8
	Merchant	69	11.4
	House wife	286	47.2
	Farmer	67	11.1
	Others	46	7.6

Monthly income in Birr	<5000	419	69.2
	>5000	187	30.8

Client's husband's educational status (^*∗*^)	Cannot read and write	137	22.9
	Primary school	120	20.1
	Secondary school	147	24.6
	College or university	193	32.3

Client's husband's occupation (^*∗*^)	Government employee	172	28.8
	Self-employed	70	11.7
	Merchant	129	21.6
	Farmer	157	26.3
	Others	69	11.6

**Table 2:** Description of previous and current obstetrics and medical-related factors of study participants in Felege Hiwot Referral Hospital, June–August 2018 (n = 606).

Variables	Category	Number	Percent
Parity	Nulliparous	244	40.3
	I–IV	291	48
	≥V	71	11.7

Gestational age in weeks	<34	46	7.6
	34–36^+6^	60	9.9
	37–38^+6^	182	30
	39–40^+6^	215	35.5
	41–41^+6^	46	7.6
	≥42	57	9.4

ANC follow-up	Yes	577	95.2
	No	29	4.8

Number of antepartum complications	None	309	51
	One	211	34.8
	Two and above	86	14.2

Antepartum hemorrhage	Yes	51	8.4
	No	555	91.6

Premature rupture of the membranes	Yes	98	16.2
	No	508	83.8

Oligohydramnios	Yes	57	9.4
	No	549	90.6

Intrauterine growth restriction	Yes	49	8.1
	No	557	91.9

Pregnancy-induced hypertension	Yes	136	22.4
	No	470	77.6

Duration of labor >15 hrs	Yes	181	29.9
	No	425	70.1

Induction or augmentation	Yes	113	18.6
	No	493	81.4

Nonreassuring fetal heart rate pattern	Yes	74	12.2
	No	532	87.8

Previous cesarean scar	Yes	99	16.3
	No	507	83.7

Interpregnancy interval <2 years	Yes	60	9.9
	No	546	90.1

Interpregnancy interval >5 years	Yes	73	12
	No	533	88

Previous history of perinatal death	Yes	62	10.2
	No	544	89.8

Chronic hypertension	Yes	13	2.1
	No	593	97.9

Maternal anemia	Yes	71	11.7
	No	535	88.3

**Table 3:** Description of labor and delivery outcome variables of study participants in Felege Hiwot Referral Hospital, June–August 2018 (*n* = 606).

Variables	Meconium-stained amniotic fluid		Clear amniotic fluid	
	Number	Percent	Number	Percent
Mode of delivery				
Spontaneous vaginal delivery	55	36.9	261	57.1
Instrumental delivery	10	6.7	19	4.2
Cesarean section	84	56.4	177	38.7

Newborn sex				
Male	77	51.7	239	52.3
Female	72	48.3	218	47.7

Birth weight in kilograms				
Less than 2.5	32	21.5	104	22.8
≥2.5	117	78.5	353	77.2

First minute Apgar score				
<7	52	34.9	35	7.7
≥7	97	65.1	422	92.3

Fifth-minute Apgar score				
0	3	2.0	6	1.3
1–3	0	0	0	0
4–6	11	7.4	6	1.3
≥7	135	90.6	445	97.4

Newborn resuscitated				
Yes	72	48.3	41	9.0
No	77	51.7	416	91.0

Newborn intubated				
Yes	16	10.7	10	2.2
No	133	89.3	447	97.8

Referral to the NICU				
Yes	80	53.7	104	22.8
No	69	46.3	353	77.2

Reason for referral				
Low birth weight	6	7.4	31	29.8
Perinatal asphyxia	7	8.6	5	4.8
Meconium aspiration syndrome	45	55.6	0	0
Early-onset neonatal sepsis	12	14.8	22	21.2
Respiratory distress syndrome	4	4.9	10	9.6
Others	7	8.6	36	34.2

Proportion	149	24.9	457	75.4

**Table 4:** Maternal factors associated with meconium-stained amniotic fluid, bivariate and multivariable logistic regression analysis, Felege Hiwot Referral Hospital, Ethiopia, 2018.

Variables		Meconium-stained amniotic fluid		COR (95% CI)	*p* value	AOR (95% CI)	*p* value
		Yes, *N*(%)	No, *N*(%)				
Client educational status	Illiterate	53 (8.7)	121 (20.0)	1.90 (1.14, 3.19)	.015	0.88 (0.43, 1.79)	.723
	≥Primary school	96 (15.9)	210 (34.6)	1		1	

Monthly income in Birr	<5000	115 (19)	304 (50.2)	1.71 (1.11, 2.62)	.015	2.03 (1.18, 3.51)	.011^*∗*^
	≥5000	34 (5.6)	153 (25.2)	1		1	

Parity	Nulliparous	70 (11.6)	174 (28.7)	1			
	≥ one	79 (13)	283 (46.7)	0.42 (0.24, 0.74)	.003	0.62 (0.34, 1.11)	.107

ANC follow-up	Yes	138 (22.8)	439 (72.4)	1		1	
	No	11 (1.8)	18 (3.0)	1.94 (0.90, 4.22)	.092	0.86 (0.29, 2.52)	.781

Antepartum complications	Yes	78 (12.9)	219 (36.1)	1.99 (1.19, 3.31)	.009	0.70 (0.30, 1.67)	.417
	No	71 (11.7)	238 (39.3)	1		1	

Oligohydramnios	Yes	21 (3.5)	36 (5.9)	1.92 (1.10, 3.40)	.026	2.53 (1.25, 5.12)	.010^*∗*^
	No	128 (21.1)	421 (69.5)	1		1	

Fetal growth restriction	Yes	17 (2.8)	32 (5.3)	2.76 (1.52, 5.01)	.001	2.54 (0.88, 7.29)	.085
	No	132 (21.8)	425 (70.1)	1		1	

Postterm pregnancy	Yes	31 (5.1)	26 (4.3)	4.34 (2.49, 7.62)	<.001	4.54 (2.24, 9.20)	<.001^*∗*^
	No	118 (19.5)	431 (71.1)			1	

Pregnancy-induced hypertension	Yes	36 (5.9)	100 (16.5)	2.28 (1.51, 3.44)	<.001	2.43 (1.45, 4.05)	.001^*∗*^
	No	113 (18.6)	357 (58.9)	1		1	

Duration of labor >15 hrs	Yes	75 (12.4)	106 (17.5)	3.36 (2.28, 4.95)	<.001	2.83 (1.76, 4.53)	<.001^*∗*^
	No	74 (12.2)	351 (57.9)	1		1	

Induction or augmentation	Yes	36 (5.9)	77 (12.7)	1.57 (1.01, 2.46)	.048	1.20 (0.67, 2.14)	.543
	No	113 (18.6)	380 (62.7)	1		1	

Nonreassuring fetal heart rate pattern	Yes	61 (10.1)	13 (2.1)	23.68 (12.47, 44.94)	<.001	21.91 (10.96, 43.83)	<.001^*∗*^
	No	88 (14.5)	444 (73.3)	1		1	

Previous cesarean scar	Yes	13 (2.1)	86 (14.2)	2.43 (1.31, 4.48)	.005	1.05 (0.47, 2.34)	.904
	No	136 (22.5)	371 (61.2)	1		1	

Interpregnancy interval <2 years	Yes	22 (3.6)	38 (6.3)	2.84 (1.65, 4.91)	<.001	2.24 (1.12, 4.51)	.024^*∗*^
	No	127 (21.0)	419 (69.1)	1		1	

Previous history of perinatal death	Yes	21 (3.5)	41 (6.8)	1.67 (0.95, 2.92)	.076	1.12 (0.48, 2.59)	.800
	No	128 (21.1)	416 (68.6)	1		1	

Maternal anemia	Yes	25 (4.1)	46 (7.6)	1.80 (1.06, 3.05)	.029	1.37 (0.66, 2.84)	.398
	No	124 (20.5)	411 (67.8)	1		1	

^*∗*^Significant variables with *p* values <0.05.

## Data Availability

All the generated data are included in the manuscript. The original data can be obtained from the first author upon request.

## Abbreviations

APGAR: Appearance, pulse rate, grimace, activity, and respiratory rate
EDHS: Ethiopian Demographic Health Survey
ENND: Early neonatal death
ETB: Ethiopian Birr
FHRH: Felege Hiwot Referral Hospital
MSAF: Meconium-stained amniotic fluid
NRFHRP: Nonreassuring fetal heart rate pattern
PNA: Perinatal asphyxia
RDS: Respiratory distress syndrome
TTN: Transient tachypnea of the newborn
WHO: World Health Organization.
